# Risk Factors associated with noncarious cervical lesions 

**DOI:** 10.4317/jced.61349

**Published:** 2024-07-01

**Authors:** Gilsara-Araújo-Albuquerque Fontelle, Francisco-Yuri-Carneiro do Nascimento, Paulo-Goberlânio-de Barros Silva, Juliana-Paiva-Marques-Lima Rolim

**Affiliations:** 1 Post-graduate of Christus University Center (UNICHRISTUS), Fortaleza, Brazil; 2 Specialist in restorative dentistry of Christus University Center (UNICHRISTUS), Fortaleza, Brazil; 3 Dentistry professor of the Christus University Center (UNICHRISTUS), Fortaleza, Brazil

## Abstract

**Background:**

Noncarious cervical lesions (NCCLs) is the dental structure loss unrelated to caries. The aim was to investigate the possible relationship between bruxism, age, gender, daily routine and dietary habits and NCCL, and correlate with sensitivity and position in the dental arch.

**Material and Methods:**

245 patients aged between 18 and 40 participated in the survey, in which a questionnaire focused on parafunctional habits, erosion, abrasion, dental abfraction and acidic diet was conducted. Facial symmetry analysis, masseter hypertrophy, occlusion evaluation, and presence of wear facets and NCCL. Data were expressed as absolute and percentage frequency and were analyzed using the Fisher’s Exact/Chi-square tests and a multinomial logistic regression model.

**Results:**

NCCLs was observed in 46.6 % in the participants. Bruxism was observed in 64% of the individuals, and stress in 33%. Wear facets and acidic diets were reported in 75% and 60% of the sample, respectively. There was no significant relationship between NCCL and gender (p = 0.74), bruxism (*p* = 0.33), stress (*p* = 0.52), wear facets (*p* = 0.73), and acidic diets (*p* = 0.39). Age over 30 years was more strongly associated with NCCL (*p*< 0.001).

**Conclusions:**

Age showed a direct correlation with noncarious cervical lesions. Factors including gender and dietary and parafunctional habits, such as bruxism, were not differential regarding the presence of NCCL.

** Key words:**Tooth wear, non-carious cervical lesions, bruxism.

## Introduction

Noncarious cervical lesions (NCCLs) comprise a pathological process that is described by the loss of hard tissue near the cementum-enamel junction, which is unrelated to dental biofilm and caries processes (1). The dental structures of the cervical region are more vulnerable to wear since the enamel is significantly thin in this area (2). Morphologically, NCCLs are characterized by two distinct patterns: wedge-shaped lesions with acute internal angles and disc-shaped lesions with wider, rounded angles, each of which allegedly depends on etiology. The former type of NCCL is caused by abrasive factors while the latter, denominated erosion, are generally shallow and caused by acid erosion (3). Both lesions may range from superficial depressions to ample defects (4).

NCCL constitutes a chronic condition and has been positively correlated with age in some studies, in addition to brushing, nutritional and parafunctional habits (3,5,7-). Epidemiologically, pre-molars are the most affected teeth (60%), possibly due to their position in the dental arch (8).

The etiology of NCCLs is multifactorial and can be caused by the combination of different processes, including abrasion, erosion, and possibly abfraction (9). Lee and Eakle (10) first proposed the condition referred to as “abfraction”. They presented a tensile stress hypothesis, stating that, in case of traumatic occlusion, as in bruxism, excessive flexural stresses may lead to severe flexing forces, resulting in compression on one side of the tooth, and tension on the opposite side. It is well known that the enamel is prone to fractures in stressed points, producing wedge-shaped lesions at acute angles at these sites. The authors proposed that the position of these lesions depends on the direction of the lateral forces, and their size on the magnitude of the force (10). Considering that occlusal forces can cause tension concentration, occlusal interference, and premature contact, bruxism, and tightening could contribute to the etiology of NCCLs (11). Micro-cracks also render the tooth more vulnerable to erosion and/or abrasion (4). During erosion, the action of dietary or stomach acids chemically remove hard dental tissues, layer by layer (12). In abrasion, on the other hand, such removal occurs by objects or substances that come in frequent contact with the tooth surfaces, resulting in mechanical wear (13).

The bruxism, currently in healthy patients, is not considered a disorder but a behavior that may be a risk factor for some clinical consequences (14). However, some authors have currently stated that the association between NCCL and occlusal forces does not represent a direct cause and effect relationship (6).

Several controversial outcomes have been reported (15), which fail to establish any significant relationship between NCCLs and premature contact in centric occlusion. Piotrowski *et al*. (16) also did not observe significant differences in occlusal contact between subjects with NCCLs and control patients.

Therefore, the objective of the present study was to determine whether there is a relationship between bruxism age, gender, daily routine, medical history, and dietary habits and NCCls. These data are essential to define the causal factors specific to the condition and can be used to guide the implementation of preventive and treatment strategies.

## Material and Methods

This analytical, transversal, observational, descriptive and quantitative study was performed after acceptance by ethics committee and all participants signed an informed consent form. In view of the non-existence in environment of cross-sectional studies of NCCL`s prevalence in the population, Considering an infinite population with a power of 90% and a level of significant of 95%, an extrapolated N of 245 sample units was reached. The calculation was performed using the Epi Info TM , version 7.2 (Center for Disease Control and prevention-CDC, Atlanta). The final sample was 245 patients, who sought out the UNICHRISTUS odontological school clinic, aged from 18 to 40 yr, from February 2022 to August 2023. The individuals met the following inclusion criteria: complete dentition, not necessarily with the presence of third molars, absence of white patches, caries or cervical restorations, and lack of prostheses or orthodontic treatment in progress.

The patients initially underwent a clinical evaluation, which consisted of an extra oral clinical examination and an intraoral investigation to detect NCCLs and/or signs of bruxism, and the identification of wear facets. In the extra oral examination, facial symmetry and tonicity of the masseter muscle were analyzed.

A single examiner performed all the exams (associate professor) aided by a student, in a dental chair with examination light, dental mirror plan n° 5 (Hu-friedy, Framkfurt, Germany) and periodontal such probe calibrated in millimeters, Williams (Hu-friedy, Framkfurt, Germany). Each probe was utilized on twenty patients, after which, they were replaced. The vestibular, palatine and lingual sides of all teeth were examined. During the examination, the probe tip was inserted into the gingival sulcus beyond the Enamel Cement Joint (ECJ) and positioned perpendicularly to the tooth surface. The examiner moved the tip of the probe perpendicular to the surface of the tooth from the bottom of the gingival sulcus to the half the coronal height. If any irregularities were detected, they were considered a noncarious cervical lesion. The lesions were recorded in a clinical file according to the degree of severity, per tooth and surface. The degree of severity of the lesion was determined according to the TWI (Tooth Wear Index) (17), which measures the level of wear in scores from 0 to 4, with 0 being no wear, and 4 representing severe wear or pulpal exposure. The severity of the individual was calculated by summing the scores of all teeth exhibiting NCCLs.

The clinical diagnosis of bruxism was concluded after observing several signs, such as periodontal changes, abnormal tooth mobility, stiffness of the tongue and cheek mucosa, increased temporal and masseter size, mandibular deviation upon mouth opening, limited mouth opening, and abnormal tooth wear, in addition to the formation of bone exostosis.

Subsequently, the individuals were subjected to a two-part structured questionnaire. The first portion contained information related to patient identification, and the second aimed at identifying habits. Habits present in their daily routine and covering issues regarding profession: working hours, sleep quality, medication use, addictions, oral hygiene care (questions relating to the technique, force and frequency of toothbrushing), oral and dietary habits (consumption of acidic foods and beverages). Other points were also investigated, such as: presence of gastric-esophageal reflux, teeth grinding, pain in the TMJ (Temporomandibular Joint), and occurrence of headaches, in order to detect associations with varying degrees of tooth wear. An occlusal parameters (Angle´s malocclusion, overjet, overbite and crossbite) dynamic parameters (maximum intercuspation contacts, interferences in excursive movements and occlusal guides for protrusion and lateral mandibular movements) and attrition (occlusal wear). Occlusal wear was assessed by direct visual inspection of occlusal/incisal surface of teeth by examiner.

According to the responses of the questionnaire and results of clinical and occlusal examination, a data matrix was made independent variables.

The data were expressed as absolute and percentage frequency and were analyzed using the Fisher’s Exact/Chi-square tests. Additionally, a multinomial logistic regression model was employed as a form of multivariate analysis for the association of factors that independently interfered in the frequency of noncarious cervical lesions.

All analyses were performed using the Statistical Package for Social Sciences (SPSS) version 17.0 for Windows, adopting a confidence interval of 95%.

## Results

NCCLs was observed in 46.6 % in the participants. Bruxism was observed in 64% and stress in 33% of the sample. Wear facets in 75% and acid feed in 60% of the sample. There was no significant association between the presence of NCCL and gender (*p*=0.74), bruxism (*p*=0.33), stress (*p*=0.52), wear facets (*p*=0.73) and acidic diet (*p*=0.39). Age over 30 years was more strongly associated with NCCL (*p*<0.001) ( Table 1, Table 2, Table 3).

## Discussion

The present study clinically evaluated the relationship between the presence of NCCL and bruxism, gender, stress, acidic diet, sensitivity, also relating to the presence of wear facet. A prevalence of 46.6% of the NCCL was observed in this study. A recent systematic review corroborates this research, an overall prevalence of 46.7%, ranging from 9.1% to 93%, was reported (18,19). Such prevalence is relative, given, in the literature; a significant discrepancy has been verified, ranging from 35% (20), to 85% (21). Result divergence may be justified by the application of different methodologies, diagnostic criteria, geographic areas, and cultures and habits (21).

Teixeira *et al*., 2020 (18) also showed that studies with population younger than 30 years present a lower prevalence than those with populations older than 30 years. Search-like data which 91.4% of patients over 30, presented NCCLs. This outcome can be justified by the period in which older people’s teeth are exposed to the etiological factors (20,22).

Another factor associated with the lesions was sensitivity, which presented a positive correlation, corroborating with studies that correlate sensitivity with the presence of cervical lesions. Such an outcome can be attributed to dentin exposure being the primary factor present in cervical lesions, consequently, the dentin tubules become exposed to the oral environment, leading to sensitivity (23). The stereomicroscopic examination demonstrated the existence of a small loss of substance in the dental cervical area, which exposes the dentinal tubules to the action of external stimuli with the appearance of pain (24).

There is no consensus in the literature regarding an association between gender and the presence of NCCLs. Despite men exerting more mastigatory force, which leads to a greater occlusal preassure, making the teeth more susceptible to the development of cervical lesions (1). This study showed that was not correlation between gender and NCCLs.

Although scientific evidence proves that the onset and progression of noncarious cervical lesions has a multifactorial etiology that involves a network of mechanism interactions, in which stress concentrations, mechanical friction, and biocorrosion are associated (1,7), the occurrence of bruxism in patients in this study herein did not present a positive correlation. Similar observations were reported in the study conducted by Takehara *et al*. (22) and those of other authors who carried out clinical research and applied questionnaires (25). The absence of such a relationship should be taken into consideration since there is no substantial evidence that correlates NCCL and occlusion (6).

Corroborating previous research reports that there is evidence that the association between NCCL and occlusal forces was derived from finite element assessments and laboratory studies (1). These authors argue that mechanical stress only contributes to the intensification of acid demineralization in NCCLs. Some other studies have demonstrated a joint action between occlusal fatigue and acid erosion in a progressive cyclic process, supporting the multi-causality of NCCLs (4,26). Although many attempts have been made to determine the role of occlusion in the etiology of NCCLs, a systematic review concluded that, to date, there is no substantial evidence to support this association (6). The authors stated that the majority of the studies were cross-sectional, thus posing some difficulties in determining the relationship between NCCLs and occlusion since the latter is not static

Similarly to other study, no association was found between acidic diets and the occurrence of lesions (25). However, in laboratory study, such a relationship has been verified (26), suggesting that teeth exposed to acidic solution wear more significantly than those exposed to neutral ones.

It has already been reported in a meta-analysis that some dietary components could increase tooth wear occurrence, which is suggested as a risk factor for NCCLs (27). However this findings did not indicate an association between the consumption of acidic beverages and fruits, corroborating with the study of Demarco *et al*., 2022 (28).

The limitations of the study should be considered. Indirect parameters were used to identify bruxism in addition self-reported measure, it has certain limitation. A reported found that a combination of several factors are required to explain the presence of NCCLs and the isolated risk factors are not sufficient to explain their presence (29). The study was conducted cross-sectional, however a longitudinal design is recommended for futures investigations.

NCCLs were detected in almost half of the examined individuals and were more prevalent in over 30 years. It is important to know the risk factors and the association between them, in order to prevent and treatment non-carious cervical lesions.

## Figures and Tables

**Table 1:** Sample characterization.

	n	%
Cervical lesion		
No	131	53.4
Yes	114	46.6
Gender		
Female	174	71.0
Male	71	29.0
Bruxism		
No	87	35.5
Yes	158	64.4
Stress		
No	163	66.5
Yes	82	33.5
Wear facet		
No	60	24.5
Yes	185	75.5
Acidic diet		
No	98	40.0
Yes	147	60.0
Sensitivity		
No	125	51.0
Yes	120	49.0
Age (years)		
Under 30	130	53.1
Over 30	115	46.9
Topography of the lesioned tooth		
Anterior	34	13.8
Posterior	130	53.1
Anterior and Posterior	81	33.1
Maxilla of the lesioned tooth		
Maxilla	93	37.9
Mandible	83	33.9
Maxilla and Mandible	69	28.2

Data expressed in absolute and percentage frequency.

**Table 2:** Factors associated with noncarious cervical lesions.

	Cervical lesion	
	No	Yes	p-Value
Gender			
Female	100	74	0.743
	74.6%	66.7%	
Male	34	37	
	25.4%	33.3%	
Bruxism			
No	50	37	0.338
	30.9%	44.5%	
Yes	112	46	
	69.1%	55.5%	
Stress			
No	81	82	0.526
	62.8%	70.7%	
Yes	48	34	
	37.2%	29.3%	
Wear facet			
No	26	34	0.730
	20.6%	28.6%	
Yes	100	85	
	79.4%	71.4%	
Acidic diet			
No	59	39	0.393
	45.0%	34.2%	
Yes	72	75	
	55.0%	65.8%	
Sensitivity			
No	81*	44	0.026
	66.9%	35.4%	
Yes	40	80*	
	33.1%	64.6%	
Age (years)			
Under 30	94*	36	< 0.001
	79.0%	28.6%	
Over 30	25	90*	
	21.0%	71.4%	
Topography			
Anterior	0	34	0.620
	.0%	14.6%	
Posterior	11	119	
	100.0%	50.8%	
Anterior and Posterior	0	81	
	.0%	34.6%	
Maxilla			
Maxilla	10	83	0.426
	100.0%	35.3%	
Mandible	0	83	
	.0%	35.3%	
Maxilla and Mandible	0	69	
	.0%	29.4%	

*p< 0.05, Fisher’s Exact/Pearson’s Chi-Square test. Data expressed as absolute and percentage frequency.

**Table 3:** Multivariate analysis for the identification of independent factors associated with the presence of noncarious cervical lesions.

	p-Value	Adjusted OR	95% CI	
Gender	0.087	-	-	-
Bruxism	0.443	-	-	-
Stress	0.611	-	-	-
Wear facet	0.675	-	-	-
Acidic diet	0.480	-	-	-
Sensitivity	0.217	-	-	-
Age > 30 years	0.002	57.7	4.4	760.4

*p<0.05, Multinomial Logistic Regression. OR = Odds Ratio; 95% CI = 95% Confidence interval of the Adjusted OR. Age above 30 years increases the frequency of noncarious cervical lesions in 57.7 times, regardless of the other assessed factors.

## Data Availability

The datasets used and/or analyzed during the current study are available from the corresponding author.
